# Impact of acute kidney injury in donors on renal graft survival: a systematic review and Meta-Analysis

**DOI:** 10.1080/0886022X.2018.1535982

**Published:** 2018-11-06

**Authors:** Yi-Tao Zheng, Chen-Bao Chen, Xiao-Peng Yuan, Chang-Xi Wang

**Affiliations:** Organ Transplant Center, The First Affiliated Hospital, Sun Yat-sen University, Guangzhou, Guangdong, China

**Keywords:** Acute kidney injury, kidney transplantation, meta-analysis, systematic review

## Abstract

The acute kidney injury (AKI) of deceased donors was an important strategy to address donor shortage. This meta-analysis was conducted to explore the clinical effect of kidney transplantation from donors with AKI. PubMed, Embase, and Cochrane Library were searched through July 2017. Fourteen cohort studies, involving a total of 15,345 donors, were included. Studies were pooled, and the hazard ratio (HR), relative risk (RR), weighted mean difference (WMD), and their corresponding 95% confidence interval (CI) were calculated. The present meta-analysis showed no significant difference in allograft survival between the AKI and non-AKI groups (HR = 1.16, 95% CI = 0.99–1.37, *P*_heterogeneity_ = 0.238, *I^2^* = 21.6%) from 12 months to 120 months after kidney transplantation. However, the time of hospital stay was significantly longer (WMD = 2.49, 95% CI = 1.06–3.92, *P*_heterogeneity_ = 0.458, *I^2^* = 0%) and the incidence of delayed graft function (DGF) was significantly higher (RR = 1.76, 95% CI = 1.52–2.04, *P*_heterogeneity_ < 0.001, *I^2^* = 71.2%) in the AKI group than in the non-AKI group. We concluded that even though hospital stay time was longer and the incidence of DGF was significantly higher in the AKI group, there is no significant difference in allograft survival between the two groups.

## Introduction

Renal transplantation is the first-choice treatment for patients suffering from end-stage kidney failure [1–3]. It was estimated that a total of 16,000 patients received kidney transplants throughout the United States in 2012 [4]. The incidence of renal failure and hence the pressure on the waiting list for a kidney transplant have increased with the development of dietary and environmental triggers, which does not match the growth in available transplants. Improvement in health care accessibility and longevity has also led to an increasing number of patients with end-stage renal disease (ESRD) [5,6]. Kidney transplantation still has a big gap between supply and demand. In 2011, nearly 5000 patients died on the kidney waiting list, which means 14.6 deaths per day or about 1 every 99 min [7]. Approximately 34,000 patients are included in the waiting list each year, which means 93 patients per day or one patient every 15 min [7]. Unfortunately, less than 17,000 kidney transplants are performed each year, about 11,000 among them are from deceased donors [7].

The imbalance between donor and recipient of kidney transplantation facilitated the implementation of various strategies to increase donor pooling [8,9]. In this regard, it has been proposed to use the acute kidney injury (AKI) of deceased donors as an important strategy to address donor shortage [10–12]. AKI is an abrupt loss of kidney function that develops within seven days and is considered reversible based on rapid reversible serum creatinine concentrations if the etiology is corrected. For donors with AKI, many transplant centers are concerned if a kidney accompanied by ischemia and reperfusion injury could be well transplanted. Therefore, the abnormal terminal serum creatinine had been reported as the second most common cause of kidney refusal [13]. On the contrary, growing evidence shows that kidney donors who had AKI conditions have similar long-term renal function and graft survival rates compared with those who did not have AKI [14–16]. This systematic review and meta-analysis of published studies were performed to explore the clinical effect of kidney transplantation from donors with AKI.

## Materials and methods

The present meta-analysis was conducted according to the Preferred Reporting Items for Systematic Reviews and Meta-analysis (PRISMA) guidelines [17].

### Search strategy

The PubMed, Embase, and the Cochrane Library databases were searched for relevant studies up to July 2017, using the following terms and their combinations: ‘kidney/renal transplantation’, ‘acute kidney injury/AKI’, and ‘donor OR donors’. Two authors (Xiao-peng Yuan and Chang-xi Wang) reviewed all scanned studies and citations. There is no language restriction in literature search. Moreover, the references of the retrieved manuscripts were also manually cross-searched for further relevant publications.

### Selection criteria

The inclusion criteria were as follows: (1) studies on adult patients receiving kidney transplantation; (2) comparative studies with at least two comparison groups, in which one group received donors with AKI and another group received donors without AKI; and (3) studies reporting graft survival and other postoperative clinical outcomes. The exclusion criteria were (1) studies on animal models and (2) types of studies are abstract, letter, review, meta-analysis.

### Data extraction and quality assessment

All the available data were extracted from each study by two investigators (Xiao-peng Yuan and Chang-xi Wang) independently according to the inclusion criteria. Any disagreement was subsequently resolved by discussion with a third author (Yi-tao Zheng). The following data were collected from each study: first author name, publication year, study design, country where the research was performed, the number of patients by gender, mean age, time of follow-up, graft survival, delayed graft function (DGF), primary nonfunction, estimated glomerular filtration rate, acute rejection, and hospital stay. A 9-star system by Newcastle–Ottawa Scale was used for assessing the quality of cohort studies. The full score was 9 stars, and the high-quality study was defined as a study with ≥7 stars [18].

### Statistical analysis

Previously reported indirect methods were used for extracting the logHR and variance due to the paucity of prognostic literature, which reported these values directly [19]. These values were calculated from either the hazard ratios or 95% CIs where quoted, the log-rank *p-*values, or the Kaplan–Meier survival curves directly. The weighted mean difference (WMD) and 95% CIs were calculated for the continuous data, and the relative risk (RR) and 95% confidence intervals for the dichotomous data. The heterogeneity of the studies was assessed using the Cochran’s *Q* test and quantified by the *I^2^* statistic (*I^2^* more than 50% was considered significant). Both fixed-effects (Mantel–Haenszel) and random-effects (Der Simonian and Laird) models were used to combine the data (the random-effects model was used if heterogeneity was significant for *I*^2^ > 50%). The relative influence of each study on the pooled estimate was assessed by omitting one study at a time for sensitivity analysis. Publication bias was evaluated by visual inspection of symmetry of Begg’s funnel plot and assessment of Egger’s test (*p* < .05 was regarded as representative of statistical significance). Statistical analyses were done with the Stata software, version 12.0 (Stata Corp., TX, USA), and all tests were two sided.

## Results

### Characteristics of the studies

**Figure 1.**
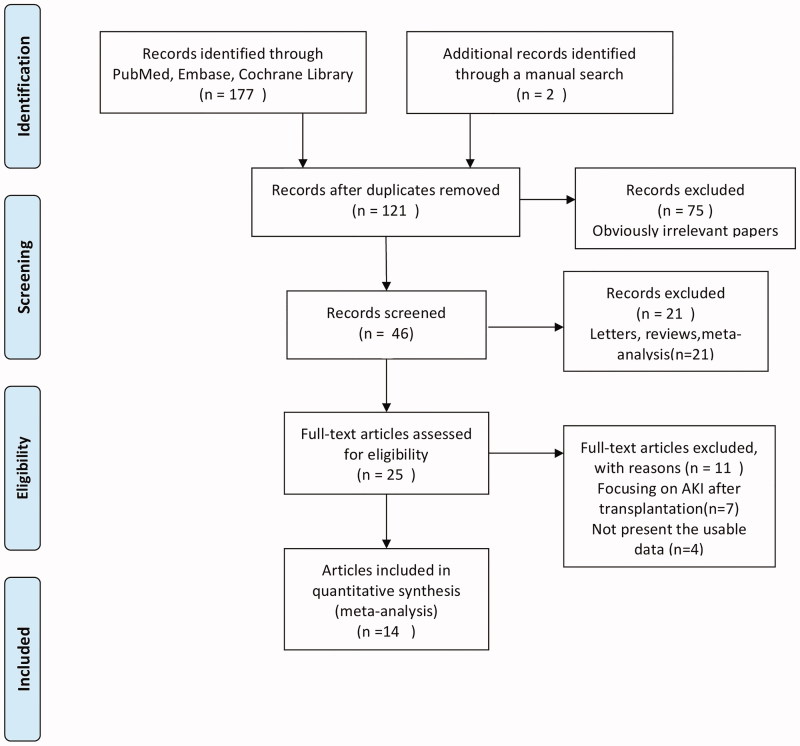
Flow diagram of identification of studies.

The initial search returned a total of 179 studies (177 from database searches and 2 from manual retrieval), of which 58 were excluded for being duplications among databases. Subsequently, 75 irrelevant studies were excluded by screening abstracts, and 21 studies were excluded by publication types of studies were letter, review, or meta-analysis. After full-text reading, 11 articles were deemed unsuitable and therefore excluded, so 14 studies [10–12,15,20–29] were identified to be included for quantitative analysis. Two of these studies [10,23] investigated donors in two different groups (standard criteria donor, SCD and expanded criteria donor, ECD), and the data were analyzed separately for each group. Therefore, they were analyzed as four subgroups. Finally, the 14 studies composed of 15,345 donors were incorporated into the present meta-analysis. The flowchart of the selection of studies and reasons for exclusion are presented in Figure 1.

The main characteristics of the eligible studies are shown in Table 1. All the included studies were cohort studies, and most of the patients were from developed countries and over 18 years old. The follow-up interval ranged from 6 to 120 months. The methodological quality of cohort studies included in the meta-analysis is shown in Table 2. For all the cohort studies are uncontrolled, and some unadjusted data extracted from Kaplan–Meier survival curves directly thus increased the risk of bias. The quality of the cohort studies included in the meta-analysis was not generally high: nine studies had six stars, and five studies had seven stars.

**Table 1.** Characteristics of the studies included in this meta-analysis.

Authors/year of publication	Country	Study design	Gender (male/female)	Mean age [year]	Donors		Follow-up [month]	Outcomes assessed
					AKI	Non-AKI		
Rodrigo/2010 [15]	Spain	Cohort study	AKI: 12/7 Non-AKI: 87/70	AKI: 46.3 ± 13.2 Non-AKI: 45.8 ± 16.7	19	157	NA	DGF, acute rejection
Kolonko/2011 [20]	Poland	Cohort study	AKI: 6/4 Non-AKI: 36/15	AKI: 50 Non-AKI: 43	10	51	49 ± 18	DGF, graft survival
Farney/2013 [21]	USA	Cohort study	AKI: 64/20 Non-AKI: 159/124	AKI: 36 ± 13 Non-AKI: 35 ± 15	84	283	6–70	DGF, graft survival, PNF
Jung/2013 [22]	Korea	Cohort study	AKI: 32/4 Non-AKI: 11/7	AKI: 45.67 ± 14.27 Non-AKI: 50.39 ± 25.18	36	18	23.2 ± 10.4	Graft survival
Jacobi /2014 (S) [23] Jacobi /2014 (E) [23]	Germany Germany	Cohort study Cohort study	AKI: 18/8 Non-AKI: 119/63	AKI: 50.4 ± 10.2 Non-AKI: 48.3 ± 11	26	182	12	DGF, graft survival, PNF, hospital stay, eGFR
			AKI:27/10 Non-AKI: 98/39	AKI: 60.1 ± 10.4 Non-AKI: 61.7 ± 10.8	37	137		
Lee/2014 [12]	Korea	Cohort study	AKI:3/11 Non-AKI: 79/34	AKI: 43.3 ± 13.8 Non-AKI: 41.1 ± 14.6	43	113	NA	DGF, graft survival, eGFR
Yu/2014 [24]	China	Cohort study	AKI: 14/5 Non-AKI: 27/11	AKI: 40 ± 9.8 Non-AKI: 35 ± 12.2	19	38	12	DGF, acute rejection, eGFR
Yuan/2014 [25]	China	Cohort study	AKI: 27/12 Non-AKI: 48/12	AKI: 37 ± 15.2 Non-AKI: 37.5 ± 13.5	29	60	7–26	DGF, acute rejection, eGFR
ALI/2015 [26]	Saudi Arabia	Cohort study	AKI: 83/18 Non-AKI: 146/14	AKI: 36.7 ± 11.0 Non-AKI: 35.0 ± 13.0	101	160	120	DGF, graft survival, acute rejection, hospital stay
Benck/2015 [27]	Germany	Cohort study	AKI: 25/8 Non-AKI: 28/37	AKI: 53 ± 13 Non-AKI: 54.8 ± 15.5	33	65	NA	DGF
Hall/2015 [11]	USA	Cohort study	AKI: 216/126 Non-AKI: 613/414	41	342	1027	20	DGF
Heilman/2015 (S) [10] Heilman/2015 (E) [10]	USA USA	Cohort study Cohort study	AKI: 108/31 Non-AKI: 288/184	AKI: 32.3 ± 13.2 Non-AKI: 34.5 ± 15.4	139	472	19.6–41.4	DGF, graft survival, acute rejection, eGFR
			AKI: 17/6 Non-AKI: 71/66	AKI:56.6 ± 9.1 Non-AKI:61.6 ± 9.2	23	137	12.3–23.8	
Boffa/2017 [28]	United Kingdom	Cohort study	NA	Over 18	1869	9350	NA	DGF, graft survival, PNF
Kim/2017 [29]	Korea	Cohort study	AKI: 53/51 Non-AKI: 103/78	AKI: 49.1 ± 11.3 Non-AKI: 46.5 ± 8.0	104	181	NA	DGF, graft survival, eGFR

AKI: Acute kidney injury; non-AKI: nonacute kidney injury; DGF: delayed graft function; PNF: primary nonfunction; eGFR: estimated glomerular filtration rate; NA: not available; S: standard criteria donor; E: expanded criteria donor.

**Table 2.** Methodological quality of cohort studies included in the meta-analysis^a^.

First author	Representativenessof the exposedcohort	Selectionof theunexposedcohort	Ascertainmentof exposure	Outcome ofinterest not presentat the start ofthe study	Control forimportant factoror additional factor	Outcomeassessment	Follow-uplong enoughfor outcomesto occur	Adequacy offollow-upof cohorts	Total qualityscores
Rodrigo/2010	⋆	⋆	⋆	⋆	—	⋆	⋆	—	5
Kolonko/2011	⋆	⋆	⋆	⋆	—	⋆	⋆	⋆	6
Farney/2013	⋆	⋆	⋆	⋆	—	⋆	⋆	⋆	6
Jung/2013	⋆	⋆	⋆	⋆	—	⋆	⋆	⋆	6
Jacobi/2014	⋆	⋆	⋆	⋆	—	⋆	⋆	⋆	6
Lee/2014	⋆	⋆	⋆	⋆	—	⋆	⋆	—	5
Yu/2014	⋆	⋆	⋆	⋆	—	⋆	⋆	⋆	6
Yuan/2014	⋆	⋆	⋆	⋆	—	⋆	⋆	⋆	6
ALI/2015	⋆	⋆	⋆	⋆	—	⋆	⋆	⋆	6
Benck/2015	⋆	⋆	⋆	⋆	—	⋆	⋆	—	5
Hall/2015	⋆	⋆	⋆	⋆	—	⋆	⋆	⋆	6
Heilman/2015	⋆	⋆	⋆	⋆	—	⋆	⋆	⋆	6
Boffa/2015	⋆	⋆	⋆	⋆	—	⋆	⋆	—	5
Kim/2015	⋆	⋆	⋆	⋆	—	⋆	⋆	—	5

a A study could be awarded a maximum of one star for each item except for the item. Control for important factor or additional factor.

### Quantitative synthesis

**Figure 2.**
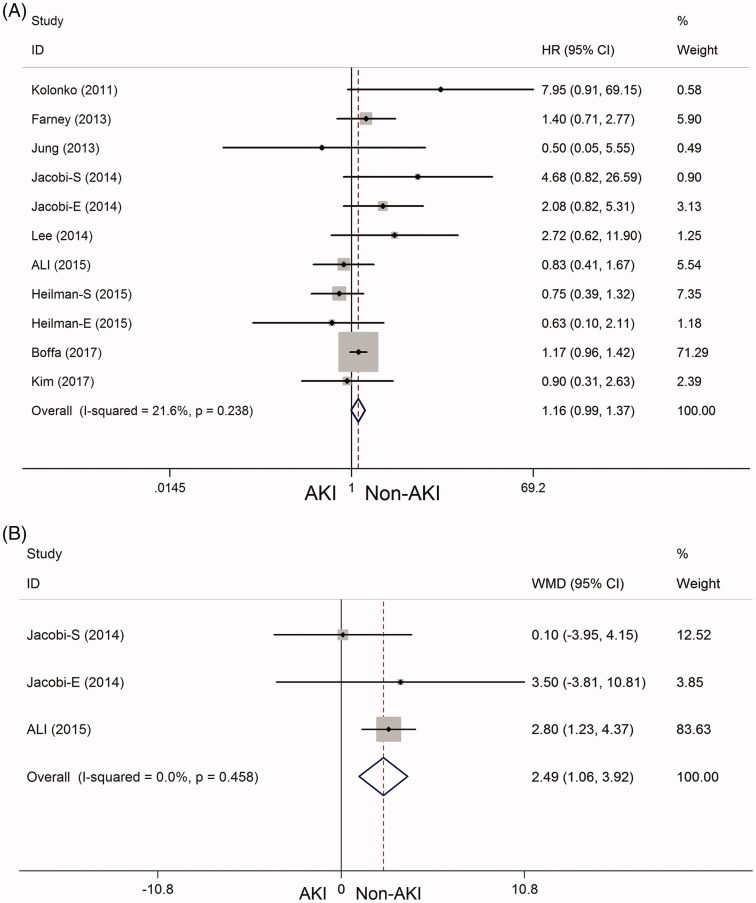
Forest plots showing the allograft survival and hospital stay after kidney transplantation in the AKI and non-AKI groups. (A) Allograft survival; (B) hospital stay.

*Allograft survival:* Nine studies [10,12,20–23,26,28,29] explored the prognostic survival benefit of kidney transplantation. Pooled analysis was performed with the available data on the correlation between AKI and allograft survival. No significant difference in allograft survival was found on comparing the AKI group with the non-AKI group (HR = 1.16, 95% CI = 0.99–1.37, *P*_heterogeneity_ = 0.238, *I^2^* = 21.6%) at 12 months to 120 months after kidney transplantation (Figure 2(A)).

*Hospital stay time:* Two studies [23,26] explored the length of hospital stay after kidney transplantation. This outcome is used to describe the duration of a single episode of hospitalization, calculated by subtracting day of admission from day of discharge. The pooled results showed that the time of hospital stay was significantly longer in the AKI group than in the non-AKI group (WMD = 2.49, 95% CI = 1.06–3.92, *P*_heterogeneity_ = 0.458, *I^2^* = 0%) (Figure 2(B)).

**Figure 3.**
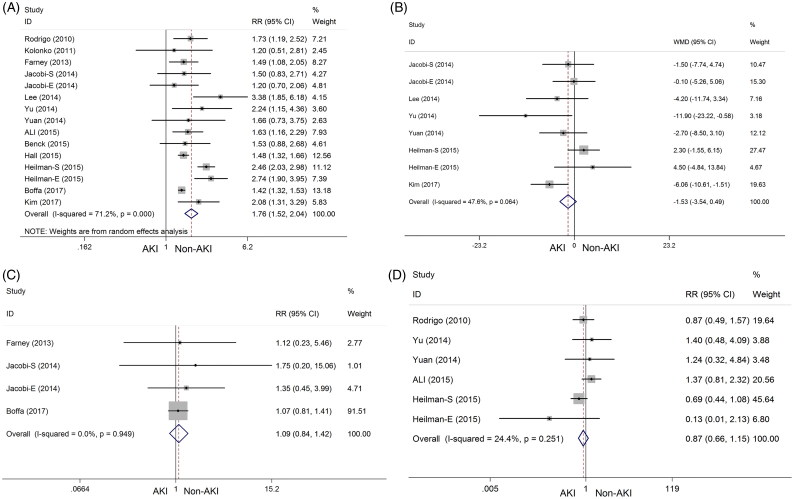
Comparison of clinical outcomes in allograft function after kidney transplantation between the AKI and non-AKI groups. (A) DGF; (B) eGFR; (C) PNF; (D) acute rejection.

*DGF*: Thirteen studies [10–12,15,20,21,23–29] explored the incidence of DGF after kidney transplantation, which was defined as at least one dialysis treatment required in the first week after transplantation [28]. The pooled results showed that the incidence of DGF was significantly higher in the AKI group than in the non-AKI group (RR = 1.76, 95% CI = 1.52–2.04, *P*_heterogeneity_ < 0.001, *I^2^* = 71.2%) (Figure 3(A)).

*Estimated glomerular filtration rate（eGFR）*: Six studies [10,12,23–25,29] explored the eGFR after kidney transplantation, which describes the flow rate of filtered fluid through the kidney. No significant difference in the improvement of eGFR at 1 year was found on comparing the AKI group with the non-AKI group (WMD = –1.53, 95% CI = –3.54–0.49, *P*_heterogeneity_ = 0.064, *I^2^* = 47.6%) (Figure 3(B)).

*Primary nonfunction (PNF)*: Three studies [21,23,28] including explored the incidence of PNF after kidney transplantation, which is also an index of kidney dysfunction. The pooled results showed no significant difference in the incidence of PNF between the two groups (RR = 1.09, 95% CI = 0.84–1.42, *P*_heterogeneity_ = 0.949, *I^2^* = 0%) (Figure 3(C)).

*Acute rejection*: Five studies [10,15,24–26] including explored the incidence of acute rejection after kidney transplantation, which is a risk of organ failure. The pooled results showed no significant difference in the incidence of acute rejection between the two groups (RR = 0.87, 95% CI = 0.66–1.15, *P*_heterogeneity_ = 0.251, *I^2^* = 24.4%) (Figure 3(D)).

### Sensitive analysis

Sensitivity analyses were performed to assess the influence of individual dataset on the pooled results by sequentially removing each eligible study. The overall statistical significance did not change when any single study was omitted, indicating that the results were statistically robust.

### Publication bias

**Figure 4.**
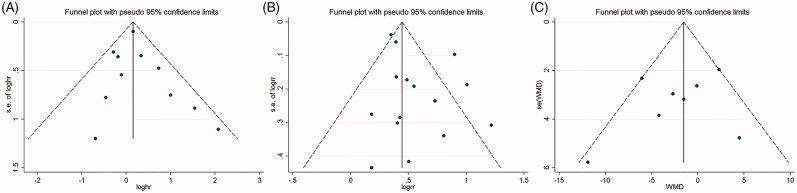
Funnel plot for publication bias test. Each point represents a separate study for the indicated association. (A) Allograft survival; (B) DGF; (C) eGFR.

Finally, the Egger’s regression test showed no evidence of asymmetrical distribution in the funnel plot in the allograft survival (Begg’s test *p* = .213; Egger’s test *p* = .482), the incidence of DGF (Begg’s test *p* = .692; Egger’s test *p* = .102), and the eGFR (Begg’s test *p* = .536; Egger’s test *p*= .411) (Figure 4).

## Discussion

AKI is frequently detected in deceased donors. It can be associated with adverse clinical outcomes in corresponding kidney transplant recipients. This novel systematic review and meta-analysis examined whether AKI in donors would impact recipient survival after renal transplantation. No significant difference in allograft survival was found on comparing the AKI group with the non-AKI group. However, the pooled results showed that the time of hospital stay was significantly longer and the incidence of DGF was significantly higher in the AKI group than in the non-AKI group. Besides, no significant difference was observed in eGFR, incidence of PNF, and acute rejection between the two groups.

Heterogeneity is a potential problem when interpreting the results of meta-analyses. In this meta-analysis, heterogeneity was found in DGF analyses. Therefore, the random-effects model was used. With sensitivity analysis, the estimated pooled RR did not change significantly, enhancing the reliability in the results of the present study. DGF is an important determinant of patient and graft survival. A complex of pathologic mechanisms intervenes in the pathophysiology of this outcome. Some factors, such as donor tissue quality, brain death and related stress, preservation variables, immune factors, and recipient variables, have an impact on DGF [30].

Transplantation remains the treatment option for patients with end-stage renal failure. The need to expand the donor pool with the increase in the number of patients has inevitably led to receiving organs, which are considered ‘marginal’. In many European national or international organ-sharing systems, the current practice is not to accept donor kidneys with AKI due to a perceived adverse outcome and goes back to dialysis after transplantation. This systematic review and meta-analysis demonstrated no significant difference in allograft survival between the AKI and non-AKI groups. The results of previous studies suggested that DGF might be a risk factor for early acute rejection [31,32]. In this study, an increased risk of acute rejection was not observed despite the higher rate of DGF in the AKI group, and the time of hospital stay was found to be significantly longer in the AKI group than in the non-AKI group.

In native kidneys, AKI severity is often associated with poor clinical outcomes, including the development of chronic kidney disease and end-stage renal disease [33]. DGF as a known risk factor of poor graft function and survival could be used in assessing the hazard of severe AKI patients undergoing kidney transplantation [34–36]. Based on prior evidence, it seems reasonable to assume that even in organ transport, donor kidney damage occurs early and completely in the ischemic period and can have some deleterious effects. Recently, Hall et al. [11] observed no detrimental effect of donor AKI on an objective measure of allograft function at 6-month post-transplant, providing reasonable and reliable evidence in support of the current clinical practice for using these kidneys with AKI.

Further, some limitations in the present meta-analysis should be acknowledged in interpreting the results. First, this systematic review and meta-analysis included only cohort studies and not all the studies described the needed information thoroughly and accurately. A second potential limitation was that the number of studies for analyzing some parameters was small, reducing the statistical power. Third, the follow-up time varied greatly among the 16 studies (ranging from 6 months to 120 months) and limited the assessment of long-term clinical effects of kidney transplantation from donors with AKI. Forth, cold ischemia time (CIT), as a risk factor of graft failure and mortality in kidney transplant recipients, was not performed a subgroup analysis for insufficient data among the included studies. Fifth, all included study provided unadjusted result. The pooled analysis of unadjusted data will be confounded by difference in AKI and non-AKI donor.

Finally, although extracted HRs and 95% CIs were extracted using the strategies reported by Tierney et al. [19], the data calculated from the Kaplan–Meier curve might not be as precise as obtaining data directly from the original study.

In conclusion, despite the limitations, this meta-analysis confirmed even though hospital stay time was longer and the incidence of DGF was significantly higher in the AKI group, there is no significant difference in allograft survival between the two groups. Further well-designed studies with larger sample size and longer period of follow-up are required to assess the long-term clinical effects of kidney transplantation from donors with AKI.
